# Impact of Microbial Leavening Agents and Fermentation Time on the In Vitro Digestibility of *Neapolitan* Pizza

**DOI:** 10.3390/foods14081418

**Published:** 2025-04-20

**Authors:** Luigia Di Stasio, Salvatore De Caro, Serena Marulo, Tiziana Di Renzo, Pasquale Ferranti, Anna Reale, Gianfranco Mamone

**Affiliations:** 1 Institute of Food Sciences, National Research Council, 83100 Avellino, Italy; salvatore.decaro@isa.cnr.it (S.D.C.); serena.marulo@gmail.com (S.M.); tiziana.direnzo@isa.cnr.it (T.D.R.); anna.reale@isa.cnr.it (A.R.); gianfranco.mamone@isa.cnr.it (G.M.); 2 Department of Agricultural Sciences, University of Naples Federico II, 80055 Portici, Italy; ferranti@unina.it

**Keywords:** digestibility, FODMAP, lactic acid bacteria, pizza, yeast

## Abstract

Baking leavening agents and fermentation conditions may influence the gastrointestinal fate of nutrients in baked goods, thereby affecting their bioavailability. This study aimed to evaluate the digestibility of sourdough pizza fermented with lactic acid bacteria species (*Levilactobacillus brevis, Fructilactobacillus sanfranciscensis, Leuconostoc pseudomesenteroides*) and yeast, compared to traditional pizza fermented with baker′s yeast. The effects of leavening time (up to 48 h) and microbial leavening agents on the nutritional profile and digestibility of baked pizzas were investigated by examining the microbiological and physico-chemical changes in the doughs, with a particular focus on the sugar content. Additionally, the degree of protein hydrolysis and the levels of FODMAPs (fermentable oligosaccharides, disaccharides, monosaccharides, and polyols) were quantified on the cooked pizzas both before and after in vitro gastrointestinal digestion. In vitro protein digestibility was not significantly influenced by the type of microbial leavening agent used or fermentation time. However, extended fermentation, particularly with lactic acid bacteria sourdough, resulted in a notable decrease in FODMAPs, thereby enhancing the digestibility and overall health profile of the pizza for individuals sensitive to these compounds. Future research should further explore the mechanisms behind these changes and their implications for dietary recommendations.

## 1. Introduction

Advances in food processing technologies have long focused on enhancing product quality, such as flavor and shelf-life, while minimizing adverse reactions to certain food components. Various processing methods can modify both the quantity and molecular structure of these components, influencing their interaction with the intestinal epithelium and the immune system [1]. Among these methods, sourdough fermentation has gained widespread popularity in baked goods due to its ability to improve the nutritional profile, degrade potentially harmful compounds, reduce anti-nutritive factors, and enhance nutrient digestibility [1,2]. A significant example involves rapidly fermentable unabsorbed carbohydrates, known collectively as FODMAPs (fermentable oligosaccharides, disaccharides, monosaccharides, and polyols), which are indigestible carbohydrates that can trigger adverse gastrointestinal reactions. Cereals, such as wheat, rye, and barley, are notable sources of FODMAPs, which include oligosaccharides (e.g., fructans and oligosaccharides of the raffinose family), disaccharides (lactose), monosaccharides (fructose), and polyols (sorbitol and mannitol). In wheat, fructans, particularly fructo-oligosaccharides (FOS), which occur at high levels, are the primary FODMAP components [3]. While these compounds are associated with gastrointestinal disorders, such as irritable bowel syndrome (IBS) [4], they also provide beneficial effects on the gut microbiota due to their dietary fiber content and prebiotic activity [5]. The beneficial effects of FODMAPs as prebiotics overlap with their harmful effects in individuals affected by IBS. Previous studies have shown that adverse reactions to FODMAPs occur when the dose exceeds 0.3 g per kg of body weight [6].

Digestive enzymes play a critical role in catalyzing the breakdown of complex macromolecules along the gastrointestinal tract, potentially influencing the hydrolysis of FODMAPs [7,8]. This process can offer health benefits by reducing their fermentation in the gut, thereby alleviating symptoms in IBS patients. Consequently, digestive enzymes targeting FODMAPS may represent a promising therapeutic tool for managing IBS symptoms while maintaining gut microbiota balance. However, scientific studies on this topic remain limited.

The effect of sourdough fermentation on FODMAP levels remains poorly explored [9], whereas its role in proteolytic activity has been more thoroughly studied [10,11]. In wheat sourdough fermentation, oligopeptides are primarily released by wheat endoproteases during the initial stage of proteolysis, while the action of lactic acid bacteria (LAB) proteases and peptidases contributes to the release of smaller peptides and free amino acids [11]. However, these effects are strain-specific, as highlighted by various authors [12,13]. Reale et al. (2021) [10] reported that sourdough fermentation impacts protein digestibility by balancing the formation and degradation of oligopeptides, a process mediated by wheat proteases and, to some extent, LAB proteases. Specifically, LAB proteases directly and indirectly degrade certain proteins, such as β-amylase, serpins, ATI, and oleosin, with a lesser effect on gluten proteins, thereby improving the digestibility of wheat-derived foods. The inactivation of β-amylase at low pH levels during sourdough fermentation may contribute to the reduction of FODMAPs in baked products, as observed in prolonged fermentation with selected LAB strains [14]. Recently, Menezes et al. (2019) [9] demonstrated that incorporating sourdough starters into bread-making not only stimulates the synthesis of organic acids, but it also affects the FODMAP content, thereby enhancing the nutritional value and sensory qualities of the final product.

This study aimed to investigate the effect of model sourdough fermentation on the properties of pizza, one of the most widely consumed food worldwide. Sourdough, traditionally used in pizza making, is well-known for enhancing palatability, sensory/quality, and digestibility [15]. The primary objective was to compare microbiological and physico-chemical properties of pizza fermented with model sourdough to those of pizza made with traditional baker’s yeast. Sourdough was produced with three selected LAB species: *Levilactobacillus brevis*, *Fructilactobacillus sanfranciscensis*, and *Leuconostoc pseudomesenteroides*. The pH, total titratable acidity, organic acids, and microbiological parameters of the pizza dough were evaluated. Additionally, the degree of protein hydrolysis and FOS content in pizza samples were analyzed before and after simulated in vitro digestion (INFOGEST model) at various leavening times (0 h, 4 h, 8 h, 16 h, 24 h, and 48 h).

## 2. Materials and Methods

### 2.1. Chemicals and Pizza Dough Ingredients

The reagent for the 2,4,6-trinitrobenzenesulfonic acid (TNBS) assay was provided by Thermo-Fischer Scientific (Bremem, Germany). Digestive enzymes and chemicals used for the preparation of digestion buffers were purchased from Sigma Aldrich (St. Louis, MO, USA).

The wheat flour used for the experiments was a refined commercial “00” type), kindly provided by Mulino Caputo (Antimo Caputo, Srl, Naples, Italy), whose proximate composition was 70% carbohydrates, 13% protein, 12% moisture, 3% fiber, 1.5% lipids, and 0.5% ash. Fresh baker′s yeast and salt (common fine table food-grade sodium chloride) were purchased from a local supermarket.

### 2.2. Strains and Starter Culture Preparation

In this study, three LAB strains named *Levilactobacillus brevis* A6, *Leuconostoc pseudomesenteroides* D4, and *Fructilactobacillus sanfranciscensis* SB52 were used to formulate a multi-strain starter culture for pizza dough fermentation. The choice of these strains was derived from a previous characterization of their tolerance to salinity, sucrose, ethanol, and acid and for their proteolytic activity [10,16]. In addition, these strains were combined in different mixed cultures used for the production of model breads. An analysis of the characteristics of these breads, in particular the volatile component, was used to select the mixed culture used in this manuscript.

The strains were kept as frozen stocks in 11% (*w*/*v*) reconstituted skimmed milk (Oxoid, Milan, Italy) containing 0.1% (*w*/*v*) ascorbic acid and stored in the microbial culture collection of the Institute of Food Sciences-National Research Council (Avellino, Italy).

For the starter culture preparation, each LAB strain was individually cultivated in De Man, Rogosa & Shape (MRS) broth (Oxoid) at a pH of 6.8 and 28 °C until the late exponential growth phase (~16 h). Cultured cells were recovered by simple stepwise centrifugation (12,000× *g*, 5 min, 4 °C), washed twice with sterile 20 mmol/L potassium phosphate buffer (pH 7.0) to remove residual growth medium, and used to formulate the LAB starter culture for the sourdough pizza preparation.

### 2.3. Dough and Pizza Making

Pizza dough was prepared in accordance with Commission Regulation (EU) n. 97/2010 to meet the requirements of “Neapolitan Pizza TSG”. The dough was formulated using the following percentages of ingredients: 60.35% flour, 37.72% water, 1.88% salt, and 0.04% microbial leavening agent. After kneading, the dough was left to rest for 20 min at room temperature before being divided into 250 g portions. The dough balls were leavened at 22 °C and 80% relative humidity for different leavening times (0, 4, 8, 16, 24, and 48 h). The dough was subsequently shaped and baked for 60 s in a wood oven at approximately 485 ± 30 °C.

The following two different doughs were prepared: (1) dough inoculated with the LAB multi-strain starter culture at a concentration of 6 log CFU/g and supplemented with 1 g of commercial baker’s yeast; (2) dough, used as control, inoculated only with 1 g of commercial baker’s yeast.

To assess the fermentation process, samples were collected from the newly formed dough (0 h) and at 4, 8, 16, 24, and 48 h and were subjected to physico-chemical (pH, acetic and lactic acid, and ethanol) and microbiological analyses (yeasts and LAB counts).

To evaluate the impact of fermentation on pizza digestibility, an in vitro static digestion was performed on cooked pizza samples obtained from dough fermented for 0, 4, 8, 16, 24, and 48 h. The digested samples from pizza produced with LAB multi-strain starter culture and baker′s yeast and the pizza control produced with baker′s yeast alone were analyzed to determine the degree of hydrolysis and FOS content.

### 2.4. Determination of pH, Ethanol, and Acetic/Lactic Acid of Pizza Doughs

The pH value was determined using a BASIC 20 pH-meter (Crison Instruments, Barcelona, Spain) after diluting 10 g of the dough with 90 mL of distilled water, under magnetic stirring. Ethanol, acetic acid, and lactic acid concentrations (expressed as g/L) were quantified using the RIDA^®^CUBE Assay Kits (Ethanol RCS4340, Acetic acid RCS4226, D/L-Lactic Acid RCS4240, respectively) from R-Biopharm (Melegnano MI, Italy), following the manufacturer’s instructions. The analyses of pH, ethanol, and lactic and acetic acid were carried out in triplicate.

### 2.5. Yeast and LAB Count of Pizza Doughs

For microbiological analyses, samples were prepared according to the following protocol: 10 g of dough was aseptically transferred into a sterile stomacher bag and diluted with 90 mL of sterile physiological solution (9 g/L NaCl). After homogenization with the Stomacher laboratory blender (1 min agitation, 1 min pause, 1 min agitation), each dough was serially diluted and plated in triplicate. LABs were counted on MRS Agar medium (Oxoid) supplemented with 4 mg/100 mL cycloheximide (Sigma Aldrich, St. Louis, MI, USA) after incubation at 28 °C for 72 h in anaerobic conditions (Gas Pack AnaeroGenTM, Oxoid). Total yeast populations were enumerated after incubation at 28 °C for 72 h on plates with YPD medium (10 g L^−1^ yeast extract, 20 g L^−1^ bacteriological peptone, 20 g L^−1^ dextrose, 20 g L^−1^ agar) supplemented with 50 mg/L streptomycin (Sigma-Aldrich). The results of yeast and LAB viable counts were expressed as log of colony forming units per gram (log CFU/g). Analyses were carried out in triplicate.

### 2.6. Enzymatic Sugar Determination of Pizza Dough

The content of glucose and fructose in pizza dough was determined with the enzymatic test kits Enzytec™ Liquid d-Glucose and Enzytec™ Liquid Sucrose/d-Glucose (R-Biopharm AG, Darmstadt, Germany), according to the manufacturer′s instructions. The assays were carried out in triplicate.

### 2.7. In Vitro Oral-Gastroduodenal Digestion of Pizza Samples

After baking, the samples of pizza were digested using the INFOGEST standardized static in vitro gastrointestinal digestion [17]. First, 1.25 × stocks of simulated salivary fluid (SSF), simulated gastric fluid (SGF), and simulated intestinal fluid (SIF) were prepared. Samples (1 g) were hydrated with SSF containing 1500 U mL^−1^ of salivary amylase and then digested by mincing for 2 min. This mixture was immediately incubated with SGF (50:50 *v*/*v*) containing 3300 U mL^−1^ of porcine pepsin (12.5 mg/mL) and egg lecithin liposomes (0.17 mM final concentration) at a pH of 2.7 and 37 °C for 2 h in a shaking water bath. Afterwards, pH was raised to 7.0 using 1 N NaOH. To simulate the duodenal digestion, SIF containing bile salts (10 mM in the final mixture, measured as cholic acid) and pancreatin from porcine pancreas (100 U mL^−1^ in the final mixture, based on the trypsin activity) were then added to the gastric digesta (50:50 *v*/*v*) for 2 h at 37 °C. Reaction was stopped by 5 min immersion in a boiling water bath, and then, samples were stored at −20 °C until analysis. All samples were analyzed in triplicate.

### 2.8. Evaluation of FOS Content in Pizza Samples

The FOS content (before and after the gastro-intestinal digestion of pizza samples) was determined with a K-FRUC kit (Megazyme, Bray, Ireland) according to McCleary et al. 2019 [18]. This approach measures the fructan content after hydrolysis to release D-fructose and D-glucose. High specificity recombinant enzymes (exo- and endo-inulinases and endo-levanase) were used to release these monosaccharides, which were then measured spectrophotometrically (AOAC Method 999.03; AACC Method 32.32) [19]. Briefly, after the hot extraction of FOS (considering the kit procedure for samples containing 0–10% *w*/*w* of fructan), the method involved the subtraction of fructose/glucose concentrations between the following two carbohydrate solutions: (i) the digestion of sucrose, maltose, and maltodextrans using sucrase/maltase; and (ii) the digestion of sucrose, maltose, and maltodextrans followed by fructans using fructanase or, rather, endo-levanase and exo- and endo-inulinases. The resulting fructose and glucose products were measured after the reaction with p-hydroxybenzoic acid hydrazide by spectrophotometry.

### 2.9. Degree of Hydrolysis in Pizza Samples

Proteolytic activity was determined by TNBS assay, which measured the concentration of total primary amino groups (–NH_2_) as reported by Reale et al. (2021) [10]. The assay was performed on pizza samples before and after gastroduodenal digestion. Briefly, 1 g of the sample was solubilized in 0.5 M NaCl and 150 mM sodium phosphate at a pH of 6.8 to a ratio of 1:3 (*w*:*v*) and mixed for 30 min at 21 °C. After centrifugation at 12,000× *g* for 20 min at 4 °C, 250 μL of supernatant was added to 250 μL of buffer borate and 500 μL of TNBS (%). Samples were incubated for 120 min at 37 °C. The reaction was stopped with HCl and SDS 10%. The absorbance of the solution was measured spectrophotometrically at 335 nm using Ultrospec 2100 pro (Amersham Bio-sciences, Uppsala, Sweden). The calibration curve was prepared using leucine (Leu) as a standard in a range of 0.0–1.0 mmol/L of Leu, and results were expressed as micrograms of Leucine equ/100 g of pizza. The standard was assayed under reaction conditions identical to those utilized for the samples.

### 2.10. Statistical Analysis

Statistical analyses were performed using GraphPad Prism version 9.0 (GRAPHPAD software Inc., San Diego, CA, USA). All experiments were conducted in triplicate, and results are expressed as the mean ± standard deviation (SD). Significant differences were determined using two-way ANOVA analysis, followed by Tukey’s test for multiple comparisons.

## 3. Results

Pizza dough was prepared using two different leavening methods, one with a combination of three LAB strains and baker’s yeast and another with baker’s yeast alone. The dough was subjected to different fermentation times (0 h, 4 h, 8 h, 16 h, 24 h, and 48 h) before baking in a wood oven. Hereafter, dough inoculated with both LAB and baker’s yeast is referred to as DL, while dough inoculated with baker’s yeast alone is referred to as DY. After baking, the corresponding pizzas are designated as PL (pizza with LAB and yeast) and PY (pizza with yeast only).

### 3.1. Microbiological and Physico-Chemical Analyses of Pizza Doughs

Microbiological analyses, including LAB and yeast counts, as well as physico-chemical analyses (pH, lactic and acetic acid, and ethanol content) were performed immediately after kneading (0 h) and after 4 h, 8 h, 16 h, 24 h, and 48 h of fermentation (Table 1).

The pH value is the most frequently used control parameter during the leavening process [20]. The freshly formed dough had a pH of about 6. During fermentation, the pH of the dough obtained with the LAB starter decreased from the initial 6.10 ± 0.04 to 3.80 ± 0.13 after 48 h fermentation, while the dough produced with baker’s yeast exhibited significantly lower acidification, with pH values decreasing from 6.13 ± 0.03 to 5.44 ± 0.04. Low pH values are characteristic of traditional sourdough, as also reported by other authors [2,21,22]. Furthermore, these results align with LAB count in DL samples, which increased from an initial value of ~6.28 log CFU/g to 7.54 log CFU/g after 48 h of fermentation.

Traces of ethanol were detected in the freshly formed doughs, the amount of which increased during leavening, reaching 9.87 ± 0.22 g/kg in DL and 8.87 ± 0.11 g/kg in DY at the end of 48 h of fermentation.

Lactic and acetic acids were absent in the freshly formed dough. During fermentation, both acids increased, but as expected, acid production was significantly higher in the DL samples than in those inoculated with the only baker’s yeast (DY), highlighting the vigorous fermentation activity of the starter LAB. After 48 h of fermentation, the lactic acid content in the DL sample reached 4.93 ± 0.91 g/kg, significantly higher than the DY sample, which measured 0.17 ± 0.02 g/kg. Acetic acid was produced at 48 h at levels of 0.8 g/kg and 0.37 g/kg in the DL and DY samples, respectively.

The detection of organic acids in the control samples (DY), although in very low amounts, was indicative of the presence of LAB contaminating the flour (as also evidenced by subsequent microbiological controls) or of the commercial baker’s yeast itself, as also reported by other authors [23,24]. As reported by other authors [25], the type and the quality of the cereal flour used is indeed the source of autochthonous LAB.

The DL sample at time zero had LAB and yeast values of 6.28 and 6.23 log CFU/g, respectively, confirming the amount inoculated. The DY sample, on the other hand, had yeast values of 6.20 log CFU/g and a low presence of LAB, likely resulting from flour and baker’s yeasts contamination, as mentioned above.

During leavening, the yeast count in DL and DY remained stable (from 6.23 log CFU/g to 6.28 log CFU/g). Both DY and DL samples showed constant yeast loads of around 6 log CFU/g at 48 h. On the other hands, LAB increased in all samples. In particular, in the sample produced with LAB starter (DL), the LAB count reached approximately 8.00 log CFU/g after 16 h of fermentation.

### 3.2. Determination of Sugars During Fermentation

**Figure 1:**
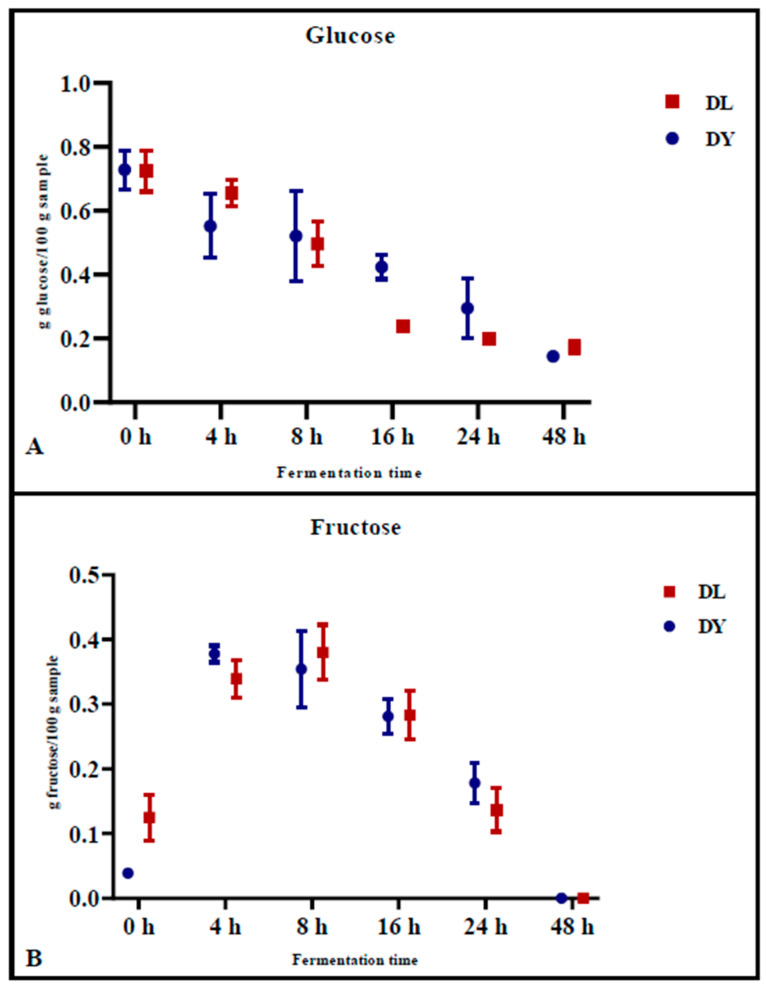
Glucose (**A**) and fructose (**B**) content in dough samples DL (mixed starter culture) and DY (control) during 48 h of fermentation. Each value represents the mean ± standard deviation (*n* ≥ 2). Statistical analysis was performed using two-way ANOVA, followed by Tukey’s test for multiple comparisons.

The levels of glucose (Figure 1A) and fructose (Figure 1B) in the doughs were monitored during fermentation. Glucose concentrations decreased similarly for both DY and DL, from 0.728 g/100 g ± 0.061 (DY) and 0.724 g/100 g ± 0.063 (DL) at time zero (0 h) to 0.144 g/100 g ± 0.002 (DY) and 0.173 g/100 g ± 0.020 (DL) after 48 h of fermentation (Figure 1A). Specifically, the reduction of glucose during fermentation was significantly higher in DL during the first 16 h of fermentation (*p* = 0.007) compared to the control dough (DY) at the same time point (*p* = 0.05) (Figure 1A). In contrast, fructose concentrations increased during the initial 8 h of fermentation, from 0.039 g/100 g ± 0.002 (DY) and 0.125 g/100 g ± 0.035 (DL) to 0.354 g/100 g ± 0.059 (DY) and 0.380 g/100 g ± 0.042 (DL), respectively, as a result of the fructans degradation. Afterward, the fructose concentration decreased to 0 after 48 h of fermentation (Figure 1B). This phenomenon could be explained by the degradation of fructans, which releases fructose during the early stage of fermentation, emphasizing the key role of LAB in the initial phase of fermentation.

### 3.3. Pizza Preparation and Simulated In Vitro Gastrointestinal Digestion: FOS Determination

**Figure 2:**
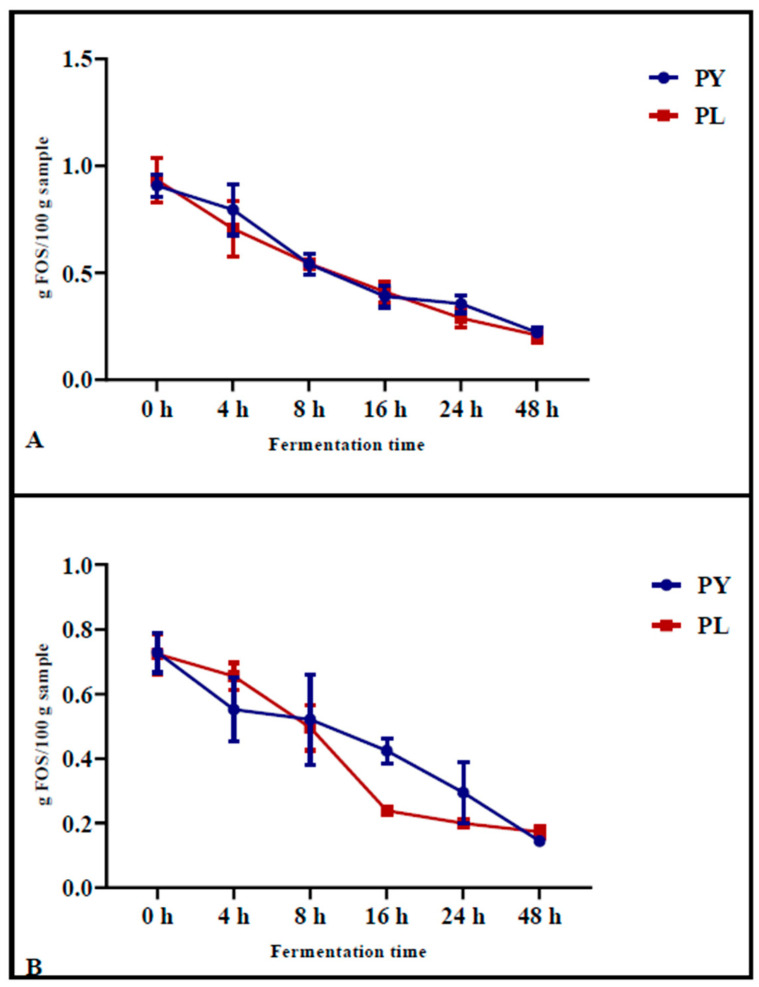
FOS content measured during 48 h of fermentation in PL (mixed starter culture) and PY (control) pizza samples before (**A**) and after (**B**) in vitro gastrointestinal digestion (INFOGEST). Each value represents the mean ± standard deviation (*n* ≥ 2). Statistical analysis was performed using two-way ANOVA, followed by Tukey′s test for multiple comparisons.

The FOS content was determined in pizza samples baked after different fermentation times (Figure 2A) and in the same samples after INFOGEST digestion (Figure 2B). The results showed that, during fermentation, the FOS content decreased progressively in both the control samples (PY) and in the sample produced with the LAB-yeast starter (PL). After 24 h of fermentation, the reduction in FOS before in vitro gastrointestinal digestion was significantly greater in PL samples (from an initial value of 0.93 g/100 g to a final value of 0.29 g/100 g, *p* < 0.0001) compared to PY (from an initial value of 0.91 g/100 g to a final value of 0.36 g/100 g, *p* = 0.0003) (Figure 2A).

Following simulated in vitro digestion, which involved gastric and pancreatic enzyme treatments, the most pronounced reduction in FOS content was observed after 16 h of fermentation in the samples previously fermented with LAB-yeast starter (Figure 2B). Specifically, in PL samples, the FOS content decreased from an initial value of 0.73 g/100 g (*p* = 0.0067) at 0 h of fermentation to a final value of 0.22 g/100 g (*p* = 0.005) at 16 h of fermentation. In contrast, in PY samples, the FOS content decreased from an initial value of 0.72 g/100 g (*p* = 0.0067) to 0.42 g/100 g (*p* = 0.05) after 16 h of fermentation (Figure 2B). These results suggest that digestion has a significant impact on the degradation of FOS and that samples fermented with the LAB starter mix undergo greater FOS degradation than the control sample after digestion.

### 3.4. Evaluation of the Hydrolysis Degree of Pizza Samples Through TNBS Assay

**Figure 3:**
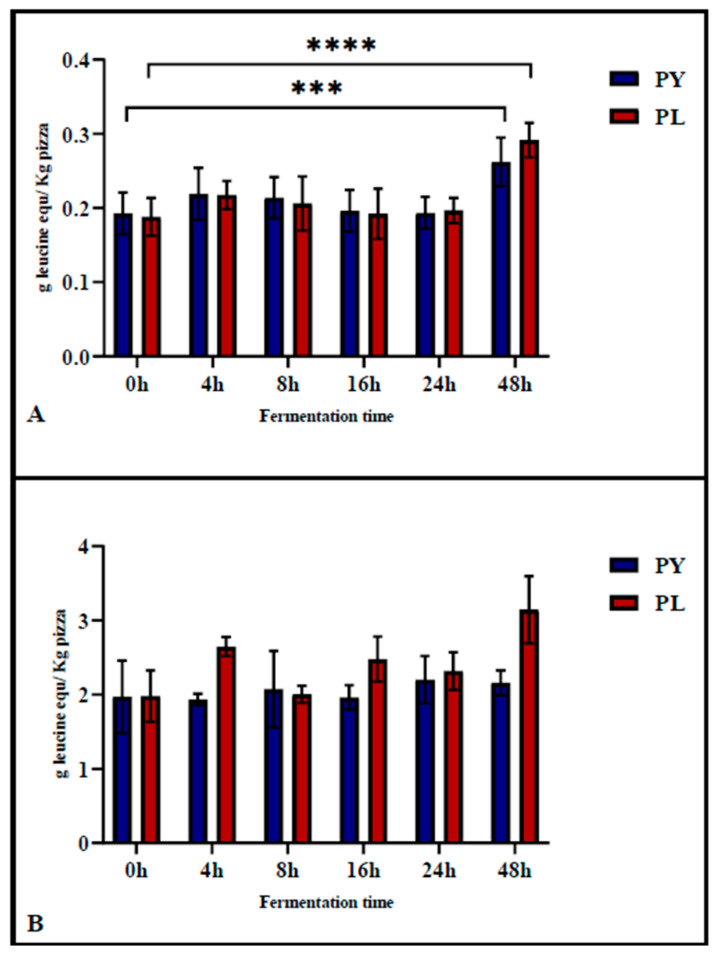
Degree of hydrolysis (free α-amino groups) in pizza samples prepared with a mixed starter culture (PL) and the control sample (PY) before (**A**) and after (**B**) in vitro gastrointestinal digestion (INFOGEST model). Results are expressed as g leucine eq/kg pizza, with each value representing the mean ± standard deviation (*n* > 3). Different superscripts (***, ****) indicate significant differences at *p* < 0.0002 and *p* < 0.0001, respectively, according to Tukey’s test for multiple comparisons.

The TNBS assay was used to determine the degree of proteolysis of the pizza before gastrointestinal digestion, assessing the proteolytic activity occurring during the fermentation phase. Subsequently, the assay was applied after in vitro gastrointestinal digestion to evaluate protein digestibility. As shown in Figure 3A, the degree of protein hydrolysis in PL and PY pizza at different fermentation times did not increase significantly during the first 24 h of fermentation. Significant differences were observed only in pizza produced after 48 h of fermentation compared to 0 h of fermentation. Notably, proteolysis at 48 h of fermentation was significantly higher in the PL sample (*p* < 0.001) compared to the PY sample (*p* = 0.004).

The digestibility of pizza proteins was evaluated using the in vitro INFOGEST model [17]. The TNBS results of digested samples (Figure 3B) showed no significant differences among pizzas fermented for different durations. This finding suggests that gastrointestinal enzyme activity had a similar impact on protein hydrolysis in both model sourdough and baker’s yeast pizza.

## 4. Discussion

Our findings highlight that extended fermentation times play crucial roles in reducing the fructans content in pizza. Under our experimental conditions, the length of fermentation significantly impacted fructans degradation. Both doughs, DY (fermented by yeasts) and DL (fermented by yeasts and LAB), reached values of about 0.3 g/100 g after 48 h of fermentation. Furthermore, during prolonged fermentation, fermentable carbohydrates such as glucose and fructose were drastically reduced. Notably, a significant decrease in glucose content was observed within the first 16 h of fermentation, particularly in DL samples. Interestingly, fructose concentrations initially increased during the first 8 h of fermentation due to fructan degradation, but they subsequently declined to near-zero levels after 48 h in both model sourdough and yeast-fermented doughs. This finding aligns with studies by Longin et al. (2020) [26] and Ziegler et al. (2016) [14], which reported a similar reduction in fructan content following prolonged fermentation. Similarly, Loponen and Gänzle (2018) [27] demonstrated that fructan hydrolysis in sourdough releases fructose, which is then partially converted into mannitol by certain LAB species. However, during extended fermentation, carbohydrate depletion can lead to the consumption of mannitol, ultimately resulting in a negligible fructose concentration in sourdough products.

Our results also showed that, after 24 h of fermentation, a significant difference in fructan content was observed between the dough fermented solely with yeast and those fermented with the model sourdough containing selected LAB and yeast. Some authors have highlighted that the synergy between LAB and yeast enhances fructan degradation [28,29]. LAB induces an acidic condition that favors yeast invertase activity, further accelerating fructan breakdown. Recent studies [10] have also demonstrated that LAB-induced fermentation degrades specific proteins, particularly β-amylase, both directly and indirectly, thereby improving the digestibility of wheat-based baked products. This process is also linked to FODMAPs degradation. Specifically, the progressive inactivation of β-amylase during fermentation, driven by protease activity and the low pH mediated by LAB, restricts the breakdown of polysaccharides into simpler sugars like maltose, which are essential for LAB fermentation [27,30]. As β-amylase becomes less active, the availability of substrates decreases, impacting the overall fermentation process. With the absence of simple sugars from starch degradation (due to β-amylase inactivation), certain microbial strains may adapt by utilizing alternative carbohydrate sources, including fructans, provided they possess the necessary enzymatic capacity. These mechanisms collectively explain the observed reduction in FODMAPs in sourdough-based baked products following prolonged fermentation facilitated by LAB [10,14].

Furthermore, our results showed that in vitro gastrointestinal digestion contributed to fructan degradation. Pizza samples obtained from doughs fermented for different leavening times were subjected to simulated digestion to evaluate their impact on fructan degradation and protein hydrolysis. The results indicated that digestion slightly reduced the fructan content in all samples. Notably, pizza produced with the model sourdough after 16 and 24 h of fermentation had a lower fructan content than those produced using yeast alone. However, the protein digestibility of pizza was shown to be unaffected by either the type of microbial leavening agent or by leavening time. In fact, the degree of protein hydrolysis after in vitro gastrointestinal digestion was similar in pizza made with sourdough and those produced using only yeasts, regardless of fermentation time.

## 5. Conclusions

In conclusion, selecting the appropriate LAB strain and optimizing fermentation parameters are critical for effectively modulating FOS hydrolysis. Extended fermentation time is particularly important, as it promotes microbial activity and the breakdown of dough constituent during leavening. In summary, the synergistic fermentation process involving sourdough and yeast is essential for FOS degradation. Future research should focus on developing low-FODMAP pizza formulations to reduce gastrointestinal discomfort while maintaining product quality.

## Figures and Tables

**Table 1:** Variation in pH values, ethanol, and lactic and acetic acid content and LAB and yeast counts in DL and DY samples at different rising times.

Time of Fermentation	Sample	pH	Lactic Acid g/L	Acetic Acid g/L	Ethanol g/L	Lactic Acid Bacteria Log CFU/g	Yeasts Log CFU/g
0 h	DL	6.10 ± 0.04	0.00 ± 0.00	0.00 ± 0.00	0.13 ± 0.01	6.28 ± 0.51	6.23 ± 0.71
	DY	6.13 ± 0.03	0.00 ± 0.00	0.00 ± 0.00	0.06 ± 0.01	3.15 ± 0.61	6.20 ± 0.60
4 h	DL	5.96 ± 0.04	0.00 ± 0.00	0.00 ± 0.00	0.43 ± 0.00	6.30 ± 0.41	5.66 ± 0.40
	DY	6.01 ± 0.06	0.00 ± 0.00	0.00 ± 0.00	0.36 ± 0.04	4.00 ± 0.31	6.15 ± 0.41
8 h	DL	6.03 ± 0.03	0.00 ± 0.00	0.09 ± 0.03	0.76 ± 0.04	6.48 ± 021	6.20 ± 0.34
	DY	6.07 ± 0.09	0.00 ± 0.00	0.00 ± 0.00	0.61 ± 0.03	4.99 ± 0.61	5.95 ± 0.20
16 h	DL	5.44 ± 0.12	0.37 ± 0.02	0.25 ± 0.11	2.11 ± 0.14	7.74 ± 0.10	6.04 ± 0.11
	DY	5.70 ± 0.05	0.00 ± 0.03	0.13 ± 0.01	3.31 ± 0.34	4.85 ± 0.11	6.11 ± 0.51
24 h	DL	4.57 ± 0.22	0.87 ± 0.40	0.51 ± 0.03	4.09 ± 0.14	8.20 ± 0.13	5.70 ± 0.32
	DY	6.02 ± 0.03	0.03 ± 0.04	0.20 ± 0.02	3.57 ± 032	5.36 ± 0.26	6.20 ± 0.44
48 h	DL	3.80 ± 0.13	4.93 ± 0.91	0.20 ± 0.02	9.87 ± 0.22	7.54 ± 0.66	6.38 ± 0.26
	DY	5.44 ± 0.04	0.17 ± 0.02	0.37 ± 0.03	8.87 ± 0.11	7.00 ± 0.56	5.48 ± 0.16

## Data Availability

The original contributions presented in the study are included in the article, further inquiries can be directed to the corresponding author.

## Abbreviations

DL	dough inoculated with LAB and baker’s yeast
DY	dough inoculated with baker’s yeast
FODMAP	fermentable oligosaccharides, disaccharides, monosaccharides, and polyols
FOS	fructo-oligosaccharides
IBS	irritable bowel syndrome
LAB	lactic acid bacteria
TNBS	2,4,6-trinitrobenzenesulfonic acid
PL	pizza with LAB and yeast
PY	pizza with yeast
SSF	simulated salivary fluid
SGF	simulated gastric fluid
SIF	simulated intestinal fluid
